# Statistical Shape Analysis of Large Datasets Based on Diffeomorphic Iterative Centroids

**DOI:** 10.3389/fnins.2018.00803

**Published:** 2018-11-12

**Authors:** Claire Cury, Joan A. Glaunès, Roberto Toro, Marie Chupin, Gunter Schumann, Vincent Frouin, Jean-Baptiste Poline, Olivier Colliot, the Imagen Consortium

**Affiliations:** ^1^Institut du Cerveau et de la Moelle épinire, ICM, Paris, France; ^2^Inserm, U 1127, Paris, France; ^3^CNRS,UMR 7225, Paris, France; ^4^Sorbonne Université, Paris, France; ^5^Inria, Aramis project-team, Paris, France; ^6^Univ Rennes, Inria, CNRS, Inserm, IRISA UMR 6074, VISAGES ERL U 1228, Rennes, France; ^7^MAP5, Université Paris Descartes, Sorbonne Paris Cité, Paris, France; ^8^Human Genetics and Cognitive Functions, Institut Pasteur, Paris, France; ^9^CNRS URA 2182 “Genes, Synapses and Cognition”, Paris, France; ^10^MRC-Social Genetic and Developmental Psychiatry Centre, Institute of Psychiatry, King's College London, London, United Kingdom; ^11^Neurospin, Commissariat à l'Energie Atomique et aux Energies Alternatives, Paris, France; ^12^Henry H. Wheeler Jr. Brain Imaging Center, University of California, Berkeley, California City, CA, United States

**Keywords:** Morphometry, shape analysis, atlas, computational anatomy, hippocampus, IHI, riemannian barycentres, LDDMM

## Abstract

In this paper, we propose an approach for template-based shape analysis of large datasets, using diffeomorphic centroids as atlas shapes. Diffeomorphic centroid methods fit in the Large Deformation Diffeomorphic Metric Mapping (LDDMM) framework and use kernel metrics on currents to quantify surface dissimilarities. The statistical analysis is based on a Kernel Principal Component Analysis (Kernel PCA) performed on the set of initial momentum vectors which parametrize the deformations. We tested the approach on different datasets of hippocampal shapes extracted from brain magnetic resonance imaging (MRI), compared three different centroid methods and a variational template estimation. The largest dataset is composed of 1,000 surfaces, and we are able to analyse this dataset in 26 h using a diffeomorphic centroid. Our experiments demonstrate that computing diffeomorphic centroids in place of standard variational templates leads to similar shape analysis results and saves around 70% of computation time. Furthermore, the approach is able to adequately capture the variability of hippocampal shapes with a reasonable number of dimensions, and to predict anatomical features of the hippocampus, only present in 17% of the population, in healthy subjects.

## 1. Introduction

Statistical shape analysis methods are increasingly used in neuroscience and clinical research. Their applications include the study of correlations between anatomical structures and genetic or cognitive parameters, as well as the detection of alterations associated with neurological disorders. A current challenge for methodological research is to perform statistical analysis on large databases, which are needed to improve the statistical power of neuroscience studies.

A common approach in shape analysis is to analyse the deformations that map individuals to an atlas or template (e.g., Ashburner et al., 1998; Chung et al., 2001; Vaillant et al., 2004; Durrleman et al., 2008; Lorenzi, 2012). The three main components of these approaches are the underlying deformation model, the template estimation method and the statistical analysis itself. The Large Deformation Diffeomorphic Metric Mapping (LDDMM) framework (Trouvé, 1998; Beg et al., 2005; Younes, 2010) provides a natural setting for quantifying deformations between shapes or images. This framework provides diffeomorphic transformations which preserve the topology and also provides a metric between shapes. The LDDMM framework is also a natural setting for estimating templates from a population of shapes, because such templates can be defined as means in the induced shape space. Various methods have been proposed to estimate templates of a given population using the LDDMM framework (Vaillant et al., 2004; Glaunès and Joshi, 2006; Durrleman et al., 2008; Ma et al., 2008). All methods are computationally expensive due the complexity of the deformation model. This is a limitation for the study of large databases.

In this paper, we present a fast approach for template-based statistical analysis of large datasets in the LDDMM setting, and apply it to a population of 1,000 hippocampal shapes. The template estimation is based on iterative diffeomorphic centroid approaches, which two of them (IC1 and IC2) were introduced at the Geometric Science of Information conference GSI13 (Cury et al., 2013), while the PW approach was introduced in a chapter of the Geometric Theory of Information book (Cury et al., 2014b). The advantage of such method is the possibility of parallelising the process, easily adding new subjects and saving computation time without losing accuracy in the template estimation. Furthermore, the iterative nature of the methodology is useful when data are being continuously collected or pre-processed. Indeed, in such cases, there is no need to recompute the mean shape each time new subjects are added to the population, the mean shape is only refined. The main idea of these methods is to iteratively update a centroid shape by successive matchings to the different subjects. This procedure involves a limited number of matchings and thus quickly provides a template estimation of the population. Iterative centroid methods have already been used to estimate mean shapes like the hippocampus (Cury et al., 2014a, 2017) and the thalamus (Cury et al., 2018). We previously showed that these centroids can be used to initialize a variational template estimation procedure (Cury et al., 2014b), and that even if the ordering of the subject along iterations does affect the final result, all centres are very similar. Here, we propose to use these centroid estimations directly for template-based statistical shape analysis. The analysis is done on the tangent space to the template shape, either directly through Kernel Principal Component Analysis (Kernel PCA Schölkopf et al., 1997) or to approximate distances between subjects. We perform a thorough evaluation of the approach using three datasets: one synthetic dataset and two real datasets composed of 50 and 1,000 subjects respectively. In particular, we study extensively the impact of different centroids on statistical analysis, and compare the results to those obtained using a standard variational template method. We will also use the large database to predict, using the shape parameters extracted from a centroid estimation of the population, some anatomical variations of the hippocampus in the normal population, called Incomplete Hippocampal Inversions and present in 17% of the normal population (Cury et al., 2015). IHI are interesting anatomical features also present in temporal lobe epilepsy with a frequency around 50% (Baulac et al., 1998), and is also involved in major depression disorders (Colle et al., 2016).

The paper is organized as follows. We first present in section 2 the mathematical frameworks of diffeomorphisms and currents, on which the approach is based, and then introduce the diffeomorphic centroid methods in section 3. Section 4 presents the statistical analysis. The experimental evaluation of the method is then presented in section 5, with the different data used in this paper.

## 2. Mathematical frameworks

Our approach is based on two mathematical frameworks which we will recall in this section. The Large Deformation Diffeomorphic Metric Mapping framework is used to generate optimal matchings and quantify differences between shapes. Shapes themselves are modeled using the framework of currents which does not assume point-to-point correspondences and allows performing linear operations on shapes.

### 2.1. LDDMM framework

Here we very briefly recall the main properties of the LDDMM setting (see Trouvé, 1998; Beg et al., 2005; Younes, 2010) for more details. The Large Deformation Diffeomorphic Metric Mapping framework allows analysing shape variability of a population using diffeomorphic transformations of the ambient 3D space. It also provides a shape space representation which means that shapes of the population are seen as points in an infinite dimensional smooth manifold, providing a continuum between shapes.

In the LDDMM framework, deformation maps φ:ℝ^3^ → ℝ^3^ are generated by integration of time-dependent vector fields *v*(*x*, .), with *x* ∈ ℝ^3^ and *t* ∈ [0, 1]. If *v*(*x, t*) is regular enough, i.e., if we consider the vector fields (*v*(·, *t*))_*t*∈[0, 1]_ in *L*^2^([0, 1], *V*), where *V* is a Reproducing Kernel Hilbert Space (RKHS) embedded in the space of *C*^1^ vector fields vanishing at infinity, then the transport equation:

(1) $$\left\{ \begin{array}{l} \frac{d\phi_{v}}{dt}(x,t)=v(\phi_{v}(x,t),t)\;\forall t\in [0,1]\\ \phi_{v}(x,0)=x\;\forall x\in {\mathbb{R}}^{3} \end{array}\right.$$

has a unique solution, and one sets φ_*v*_ = ϕ_*v*_(·, 1) the diffeomorphism induced by *v*(*x*, ·). The induced set of diffeomorphisms $A_{V}$ is a subgroup of the group of *C*^1^ diffeomorphisms. The regularity of velocity fields is controlled by:

(2) $$E(v):=\int_{0}^{1}||v(\cdot ,t)|{|}_{V}^{2}dt.$$

The subgroup of diffeomorphisms $A_{V}$ is equipped with a right-invariant metric defined by the rules: $\forall \varphi ,\psi \in A_{V}$,

(3) $$\left\{ \begin{array}{l} D(\varphi ,\psi )=D(id,\psi \circ \varphi^{-1})\\ D(id,\varphi )=\inf\{\int_{0}^{1}\left|\right|v(\cdot ,t)\;|{|}_{V}dt,\varphi =\phi_{v}(\cdot ,1)\} \end{array}\right.$$

i.e., the infimum is taken over all *v* ∈ *L*^2^([0, 1], *V*) such that φ_*v*_ = φ. *D*(φ, ψ) represents the shortest length of paths connecting φ to ψ in the diffeomorphisms group.

### 2.2. Momentum vectors

In a discrete setting, when the matching criterion depends only on φ_*v*_ via the images φ_*v*_(*x*_*p*_) of a finite number of points *x*_*p*_ (such as the vertices of a mesh) one can show that the vector fields *v*(*x, t*) which induce the optimal deformation map can be written via a convolution formula over the surface involving the reproducing kernel *K*_*V*_ of the RKHS *V*:

(4) $$v(x,t)=\sum_{p=1}^{n}K_{V}(x,x_{p}(t))\alpha_{p}(t),$$

where *x*_*p*_(*t*) = ϕ_*v*_(*x*_*p*_, *t*) are the trajectories of points *x*_*p*_, and $\alpha_{p}\left(t\right)\in {\mathbb{R}}^{3}$ are time-dependent vectors called momentum vectors, which completely parametrize the deformation. Trajectories *x*_*p*_(*t*) depend only on these vectors as solutions of the following system of ordinary differential equations:

(5) $$\begin{array}{r} \frac{dx_{q}\left(t\right)}{dt}=\overset{n}{\underset{p=1}{\sum }}K_{V}\left(x_{q}\left(t\right),x_{p}\left(t\right)\right)\alpha_{p}\left(t\right), \end{array}$$

for 1 ≤ *q* ≤ *n*. This is obtained by plugging formula 4 for the optimal velocity fields into the flow Equation 1 taken at *x* = *x*_*q*_. Moreover, the norm of *v*(·, *t*) also takes an explicit form:

(6) $$||V(\cdot ,t)|{|}_{V}^{2}=\sum_{p=1}^{n}\sum_{q=1}^{n}\alpha_{p}{\left(t\right)}^{T}K_{V}(x_{p}(t),x_{q}(t))\alpha_{q}(t).$$

Note that since *V* is a space of vector fields, its kernel *K*_*V*_(*x, y*) is in fact a 3 × 3 matrix for every *x, y* ∈ ℝ^3^. However we will only consider scalar invariant kernels of the form $K_{V}\left(x,y\right)=h\left(||x-y|{|}^{2}/\sigma_{V}^{2}\right)I_{3}$, where *h* is a real function (in our case we use the Cauchy kernel *h*(*r*) = 1/(1 + *r*)), and σ_*V*_ a scale factor. In the following we will use a compact representation for kernels and vectors. For example Equation 6 can be written:

(7) $$||V(\cdot ,t)|{|}_{V}^{2}=\alpha {\left(t\right)}^{T}K_{V}(x(t))\alpha (t),$$

where **α**(*t*) = (α_*p*_(*t*))_*p* = 1…*n*_, ∈ℝ^3 × *n*^, ***x***(*t*) = (*x*_*p*_(*t*))_*p* = 1…*n*_, ∈ℝ^3 × *n*^ and *K*_*V*_(***x***(*t*)) the matrix of *K*_*V*_(*x*_*p*_(*t*), *x*_*q*_(*t*)).

#### 2.2.1. Geodesic shooting

The minimization of the energy *E*(*v*) in matching problems can be interpreted as the estimation of a length-minimizing path in the group of diffeomorphisms $A_{V}$, and also additionally as a length-minimizing path in the space of point sets when considering discrete problems. Such length-minimizing paths obey geodesic equations (see Vaillant et al., 2004) which write as follows:

(8) $$\left\{ \begin{array}{l} \frac{dx(t)}{dt}=K_{V}(x(t))\alpha (t)\\ \frac{d\alpha (t)}{dt}=-\frac{1}{2}\nabla_{x(t)}[\alpha {\left(t\right)}^{T}K_{V}(x(t))\alpha (t)], \end{array}\right.$$

Note that the first equation is nothing more than Equation 5 which allows to compute trajectories *x*_*p*_(*t*) from any time-dependent momentum vectors α_*p*_(*t*), while the second equation gives the evolution of the momentum vectors themselves. This new set of ODEs can be solved from any initial conditions (*x*_*p*_(0), α_*p*_(0)), which means that the initial momentum vectors α_*p*_(0) fully determine the subsequent time evolution of the system (since the *x*_*p*_(0) are fixed points). As a consequence, these initial momentum vectors encode all information of the optimal diffeomorphism. For example, the distance *D*(*id*, φ) satisfies

(9) $$D{\left(id,\varphi \right)}^{2}=E(v)=||V(\cdot ,0)|{|}_{V}^{2}=\alpha {\left(0\right)}^{T}K_{V}(x(0))\alpha (0),$$

We can also use geodesic shooting from initial conditions (*x*_*p*_(0), α_*p*_(0)) in order to generate any arbitrary deformation of a shape in the shape space.

### 2.3. Shape representation: currents

The use of currents (Schwartz, 1952; de Rham, 1955) in computational anatomy was introduced by Vaillant and Glaunès (2005) and Glaunès (2005) and subsequently developed by Durrleman (2010). The basic idea is to represent surfaces as currents, i.e., linear functionals on the space of differential forms and to use kernel norms on the dual space to express dissimilarities between shapes. Using currents to represent surfaces has some benefits. First it avoids the point correspondence issue: one does not need to define pairs of corresponding points between two surfaces to evaluate their spatial proximity. Moreover, metrics on currents are robust to different samplings and topological artifacts and take into account local orientations of the shapes. Another important benefit is that this model embeds shapes into a linear space (the space of all currents), which allows considering linear combinations such as means of shapes in the space of currents.

Let us briefly recall this setting. For sake of simplicity we present currents as linear forms acting on vector fields rather than differential forms which are an equivalent formulation in our case. Let *S* be an oriented compact surface, possibly with boundary. Any smooth vector field *w* of ℝ^3^ can be integrated over *S* via the rule:

(10) $$[S](w)=\int_{S}\langle w(x),\;n(x)\rangle d\sigma_{S}(x),$$

with *n*(*x*) the unit normal vector to the surface, *dσ*_*S*_ the Lebesgue measure on the surface *S*, and [*S*] is called a 2-current associated to *S*.

Given an appropriate Hilbert space (*W*,〈·, ·〉*W*) of vector fields, continuously embedded in $C_{0}^{1}\left({\mathbb{R}}^{3},{\mathbb{R}}^{3}\right)$, the space of currents we consider is the space of continuous linear forms on *W*, i.e., the dual space W^*^. For any point *x* ∈ ℝ^3^ and vector α ∈ ℝ^3^ one can consider the Dirac functional $\delta_{x}^{\alpha }:w\mapsto \langle w\left(x\right),\alpha \rangle$ which belongs to W^*^. The Riesz representation theorem states that there exists a unique *u* ∈ *W* such that for all *w* ∈ *W*, $\langle u,w\rangle W=\delta_{x}^{\alpha }\left(w\right)=\langle w\left(x\right),\alpha \rangle$. *u* is thus a vector field which depends on *x* and linearly on α, and we write it *u* = *K_W_*(·, *x*)α. *K_W_*(*x, y*) is a 3 × 3 matrix, and *K_W_*:ℝ^3^×ℝ^3^ → ℝ^3 × 3^ the mapping called the reproducing kernel of the space *W*. Thus we have the rule

$${\langle K_{W}\left(\cdot ,x\right)\alpha ,w\rangle }_{W}=\langle w\left(x\right),\alpha \rangle .$$

Moreover, applying this formula to *w* = *K_W_*(·, *y*)β for any other point *y* ∈ ℝ^3^ and vector β ∈ ℝ^3^, we get

(11) $$\begin{array}{l} \langle K_{W}(\cdot ,x)\alpha ,K_{W}(\cdot ,y)\beta \rangle_{W}=\langle K_{W}(x,y)\beta ,\alpha \rangle \\ \;=\alpha^{T}K_{W}(x,y)\beta ={\langle \delta_{x}^{\alpha },\delta_{y}^{\beta }\rangle }_{W^{*}} \end{array}$$

Using equation 11, one can prove that for two surfaces *S* and *T*,

(12) $${\langle \left[S],[T\right]\rangle }_{W^{*}}=\int_{S}\int_{T}\langle n_{S}(x),K_{W}(x,y)n_{T}(y)\rangle \;d\sigma_{S}(x)d\sigma_{T}\left(y\right)$$

This formula defines the metric we use as data attachment term for comparing surfaces. More precisely, the difference between two surfaces is evaluated via the formula:

(13) $$||[S]-[T]|{|}_{W^{*}}^{2}={\langle \left[S],[S\right]\rangle }_{W^{*}}+{\langle \left[T],[T\right]\rangle }_{W^{*}}-2{\langle \left[S],[T\right]\rangle }_{W^{*}}$$

The type of kernel fully determines the metric and therefore will have a direct impact on the behavior of the algorithms. We use scalar invariant kernels of the form $KW\left(x,y\right)=h\left(||x-y|{|}^{2}/\sigma_{W}^{2}\right)I_{3}$, where *h* is a real function (in our case we use the Cauchy kernel *h*(*r*) = 1/(1 + *r*)), and σ_*W*_ a scale factor.

Note that the varifold (Charon and Trouvé, 2013) can be also use for shape representation without impacting the methodology. The shapes we used for this study are well represented by currents.

### 2.4. Surface matchings

We can now define the optimal match between two currents [*S*] and [*T*], which is the diffeomorphism minimizing the functional

(14) $$J_{S,T}\left(v\right)=\gamma E\left(v\right)+{\left\|\left[\varphi_{v}\left(S\right)\right]-\left[T\right]\right\|}_{W^{*}}^{2}$$

This functional is non convex and in practice we use a gradient descent algorithm to perform the optimization, which cannot guarantee to reach a global minimum. We observed empirically that local minima can be avoided by using a multi-scale approach in which several optimization steps are performed with decreasing values of the width σ_*W*_ of the kernel *K_W_* (each step provides an initial guess for the next one). Evaluations of the functional and its gradient require numerical integrations of high-dimensional ordinary differential equations (see equation 5), which is done using Euler trapezoidal rule. Note that three important parameters control the matching process: γ controls the regularity of the map, σ_*V*_ controls the scale in the space of deformations and σ_*W*_ controls the scale in the space of currents.

### 2.5. GPU implementation

To speed up the matchings computation of all methods used in this study (the variational template and the different centroid estimation algorithms), we use a GPU implementation for the computation of kernel convolutions. This computation constitutes the most time-consuming part of LDDMM methods. Computations were performed on a Nvidia Tesla C1060 card. The GPU implementation can be found here: http://www.mi.parisdescartes.fr/~glaunes/.

## 3. Diffeomorphic centroid methods

Computing a template in the LDDMM framework can be highly time consuming, taking a few days or some weeks for large real-world databases. Here we propose a fast approach which provides a centroid correctly centred among the population. Matlab codes are available here: https://github.com/cclairec/Iterative_Centroid, and use the GPU implementation mentionned above for the kernel convolutions.

### 3.1. General idea

The LDDMM framework, in an ideal setting (exact matching between shapes), sets the template estimation problem as a centroid computation on a Riemannian manifold. The Fréchet mean is the standard way for defining such a centroid and provides the basic inspiration of all LDDMM template estimation methods.

If ***x***^*i*^, 1 ≤ *i* ≤ *N* are points in ℝ^*d*^, then their centroid is defined as

(15) $$b^{N}=\frac{1}{N}\sum_{i=1}^{N}x^{i}.$$

It also satisfies the following two alternative characterizations:

(16) $$b^{N}=\underset{y\in {\mathbb{R}}^{d}}{\arg\min}\sum_{1\leq i\leq N}\|y-x^{i}{\|}^{2}.$$

and

(17) $$\left\{ \begin{array}{l} b^{1}=x^{1}\\ b^{k+1}=\frac{k}{k+1}b^{k}+\frac{1}{k+1}x^{k+1},\;1\leq k\leq N-1. \end{array}\right.$$

Now, when considering points ***x***^*i*^ living on a Riemannian manifold *M* (we assume *M* is path-connected and geodesically complete), the definition of ***b***^*N*^ cannot be used because *M* is not a vector space. However the variational characterization of ***b***^*N*^ as well as the iterative characterization, both have analogs in the Riemannian case. The Fréchet mean is defined under some hypotheses (see Arnaudon et al., 2012) on the relative locations of points ***x***^*i*^ in the manifold:

(18) $$b^{N}=\underset{y\in M}{\text{arg min}}\sum_{1\leq i\leq N}d_{M}{\left(y,x^{i}\right)}^{2}.$$

Many mathematical studies (as for example Karcher, 1977; Kendall, 1990; Le, 2004; Afsari, 2011; Arnaudon et al., 2012; Afsari et al., 2013), have focused on proving the existence and uniqueness of the mean, as well as proposing algorithms to compute it. However, these approaches are computationally expensive, in particular in high dimension and when considering non trivial metrics. An alternative idea consists in using the Riemannian analog of the second characterization:

(19) $$\left\{ \begin{array}{l} {\tilde{b}}^{1}=x^{1}\\ {\tilde{b}}^{k+1}=geod({\tilde{b}}^{k},x^{k+1},\frac{1}{k+1}),\;1\leq k\leq N-1, \end{array}\right.$$

where *geod*(***y***, ***x***, *t*) is the point located along the geodesic from ***y*** to ***x***, at a distance from ***y*** equal to *t* times the length of the geodesic. This does not define the same point as the Fréchet mean, and moreover the result depends on the ordering of the points. In fact, all procedures that are based on decomposing the Euclidean equality $b^{N}=\frac{1}{N}\overset{N}{\underset{i=1}{\sum }}x^{i}$ as a sequence of pairwise convex combinations lead to possible alternative definitions of centroid in a Riemannian setting. However, this should lead to a fast estimation. We hypothesize that, in the case of shape analysis, it could be sufficient for subsequent template based statistical analysis. Moreover, this procedure has the side benefit that at each step ${\tilde{b}}^{k}$ is the centroid of the ***x***^*i*^, 1 ≤ *i* ≤ *k*.

In the following, we present three algorithms that build on this idea. The two first methods are iterative, and the third one is recursive, but also based on pairwise matchings of shapes.

### 3.2. Direct iterative centroid (IC1)

The first algorithm roughly consists in applying the following procedure: given a collection of *N* shapes *S*_*i*_, we successively update the centroid by matching it to the next shape and moving along the geodesic flow. More precisely, we start from the first surface *S*_1_, match it to *S*_2_ and set $B_{2}=\phi_{v^{1}}\left(S_{1},1/2\right)$. *B*_2_ represents the centroid of the first two shapes, then we match *B*_2_ to *S*_3_, and set as $B_{3}=\phi_{v^{2}}\left(B_{2},1/3\right)$. Then we iterate this process (see Algorithm 1).

**Algorithm 1:**
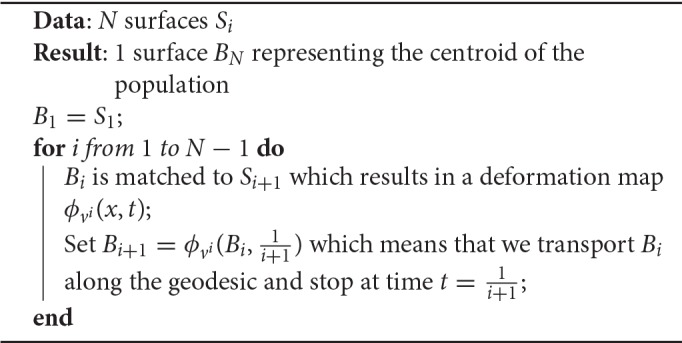
Iterative Centroid 1 (IC1)

### 3.3. Centroid with averaging in the space of currents (IC2)

Because matchings are not exact, the centroid computed with the IC1 method accumulates small errors which can have an impact on the final centroid. Furthermore, the final centroid is in fact a deformation of the first shape *S*_1_, which makes the procedure even more dependent on the ordering of subjects than it would be in an ideal exact matching setting. In this second algorithm, we modify the updating step by computing a mean in the space of currents between the deformation of the current centroid and the backward flow of the current shape being matched. Hence the computed centroid is not a surface but a combination of surfaces, as in the template estimation method. The algorithm proceeds as presented in Algorithm 2.

**Algorithm 2:**
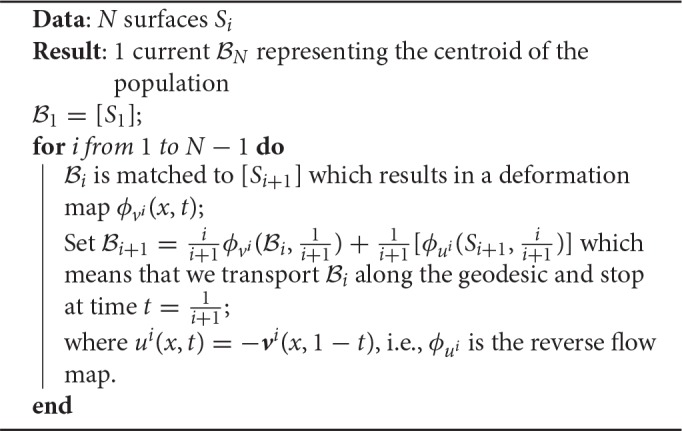
Iterative Centroid 1 (IC2)

The weights in the averaging reflect the relative importance of the new shape, so that at the end of the procedure, all shapes forming the centroid have equal weight $\frac{1}{N}$.

Note that we have used the notation $\phi_{v^{i}}\left(B_{i},\frac{1}{i+1}\right)$ to denote the transport (push-forward) of the current $B_{i}$ by the diffeomorphism. Here $B_{i}$ is a linear combination of currents associated to surfaces, and the transported current is the linear combination (keeping the weights unchanged) of the currents associated to the transported surfaces.

### 3.4. Alternative method : pairwise centroid (PW)

**Figure 1:**
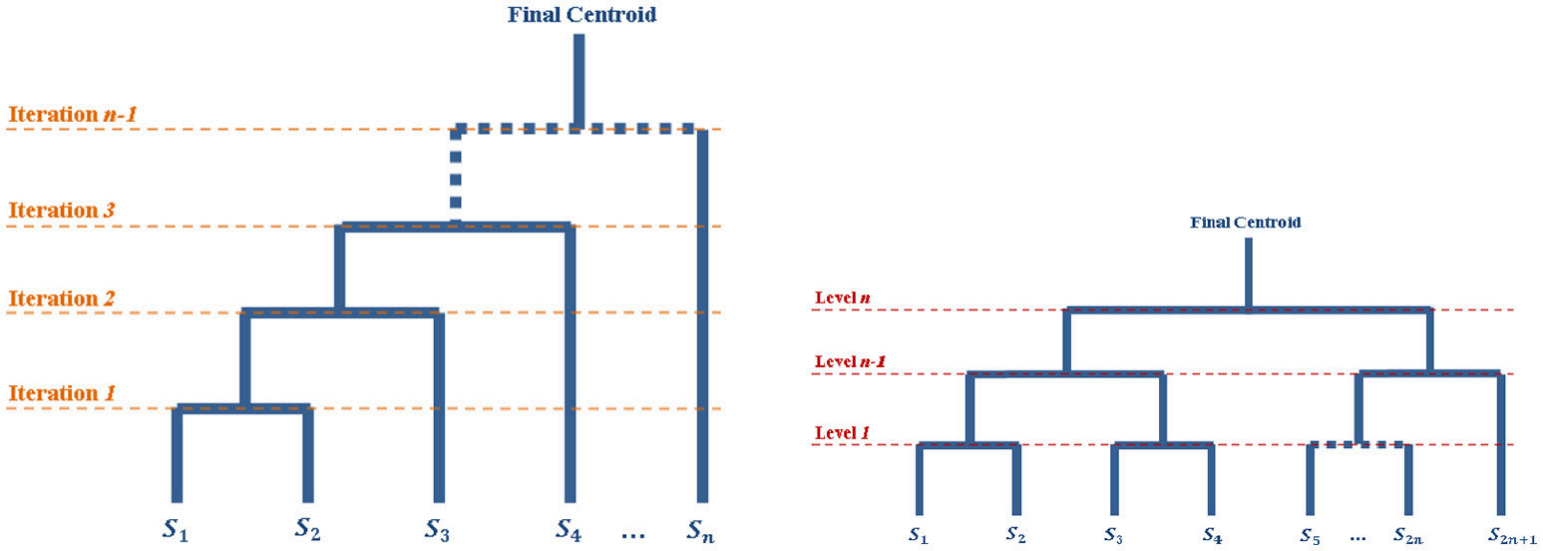
Diagrams of the iterative processes which lead to the centroids computations. The tops of the diagrams represent the final centroid. The diagram on the left corresponds to the Iterative Centroid algorithms (IC1 and IC2). The diagram on the right corresponds to the pairwise algorithm (PW).

Another possibility is to recursively split the population in two parts until having only one surface in each group (see Figure 1), and then going back up along the dyadic tree by computing pairwise centroids between groups, with appropriate weight for each centroid (Algorithm 3).

**Algorithm 3:**
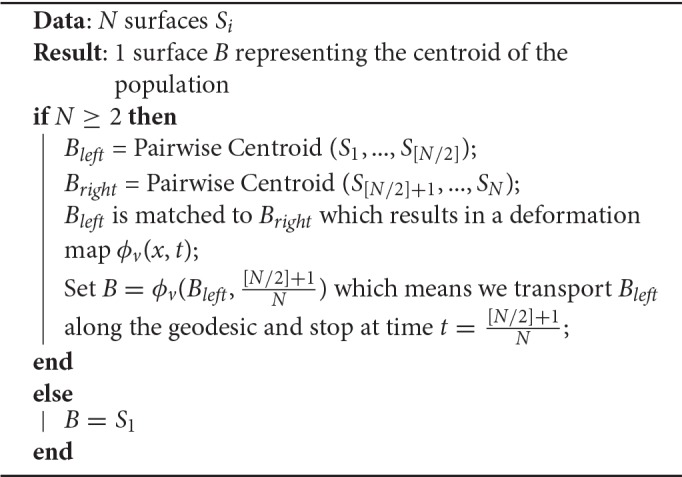
Pairwise Centroid (PW)

#### 3.4.1. Remarks on the IC algorithms

These three methods depend on the ordering of subjects. In a previous work (Cury et al., 2014b), we showed empirically that different orderings result in very similar final centroids. Here we focus on the use of such centroid for statistical shape analysis.

Regarding memory performances of the algorithm, computing a mean shape with the algorithm IC1 on meshes composed by 500 vertices, requires 1.2 MB. For the algorithm IC2, which combines surfaces at each iterations, requires more memory, for 50 meshes IC2 needs 20 MB. We used the real dataset RD50 (see section 5.1 for further details) to estimate memory performances.

### 3.5. Comparison with a variational template estimation method

In this study, we will compare our centroid approaches to a variational template estimation method proposed by Glaunès et al (Glaunès and Joshi, 2006). This variational method estimates a template given a collection of surfaces using the framework of currents. It is posed as a minimum mean squared error estimation problem. Let *S*_*i*_ be *N* surfaces in ℝ^3^ (i.e., the whole surface population). Let [*S*_*i*_] be the corresponding current of *S*_*i*_, or its approximation by a finite sum of vectorial Diracs. The problem is formulated as follows:

(20) $$\left\{\hat{v_{i}},\hat{T}\right\}=\underset{v_{i},T}{\text{arg min}}\sum_{i=1}^{N}\left\{\left\|T-\left[\varphi_{v_{i}}(S_{i})\right]{\|}_{W^{*}}^{2}+\gamma E(v_{i}\right)\right\},$$

The method uses an alternated optimization i.e., surfaces are successively matched to the template, then the template is updated and this sequence is iterated until convergence. One can observe that when φ_*i*_ is fixed, the functional is minimized when T is the average of [φ_*i*_(*S*_*i*_)]: $T=\frac{1}{N}\overset{N}{\underset{i=1}{\sum }}\left[\varphi_{v_{i}}\left(S_{i}\right)\right],$ which makes the optimization with respect to T straightforward. This optimal current is the union of all surfaces φ_*v*_*i*__(*S*_*i*_). However, all surfaces being co-registered, the ${\hat{\varphi }}_{v_{i}}\left(S_{i}\right)$ are close to each other, which makes the optimal template $\hat{T}$ close to being a true surface. Standard initialization consists in setting $T=\frac{1}{N}\overset{N}{\underset{i=1}{\sum }}\left[S_{i}\right]$, which means that the initial template is defined as the combination of all unregistered shapes in the population. Alternatively, if one is given a good initial guess T, the convergence speed of the method can be improved. In particular, the initialisation can be provided by iterative centroids; this is what we will use in the experimental section.

Regarding the computational complexity, the different centroid approaches perform *N* − 1 matchings while the variational template estimation requires *N* × *iter* matchings, where *iter* is the number of iterations. Moreover the time for a given matching depends quadratically on the number of vertices of the surfaces being matched. It is thus more expensive when the template is a collection of surfaces as in IC2 and in the variational template estimation.

## 4. Statistical analysis

The proposed iterative centroid approaches can be used for subsequent statistical shape analysis of the population, using various strategies. A first strategy consists in analysing the deformations between the centroid and the individual subjects. This is done by analysing the initial momentum vectors $\alpha^{i}\left(0\right)={\left(\alpha_{p}^{i}\left(0\right)\right)}_{p=1\ldots n}\in {\mathbb{R}}^{3\times n}$ which encode the optimal diffeomorphisms computed from the matching between a centroid and the subjects *S*_*i*_. Initial momentum vectors all belong to the same vector space and are located on the vertices of the centroid. Different approaches can be used to analyse these momentum vectors, including Principal Component Analysis for the description of populations, Support Vector Machines or Linear Discriminant Analysis for automatic classification of subjects. A second strategy consists in analysing the set of pairwise distances between subjects. Then, the distance matrix can be entered into analysis methods such as Isomap Tenenbaum et al. (2000), Locally Linear Embedding Roweis and Saul (2000), and Yang X. et al. (2011) or spectral clustering algorithms (Von Luxburg, 2007). Here, we tested two approaches: (i) the analysis of initial momentum vectors using a Kernel Principal Component Analysis for the first strategy; (ii) the approximation of pairwise distance matrices for the second strategy. These tests allow us both to validate the different iterative centroid methods and to show the feasibility of such analysis on large databases.

### 4.1. Principal component analysis on initial momentum vectors

The Principal Component Analysis (PCA) on initial momentum vectors from the template to the subjects of the population is an adaptation of PCA in which Euclidean scalar products between observations are replaced by scalar products using a kernel. Here the kernel is *K*_*V*_ the kernel of the R.K.H.S *V*. This adaptation can be seen as a Kernel PCA (Schölkopf et al., 1997). PCA on initial momentum vectors has previously been used in morphometric studies in the LDDMM setting (Vaillant et al., 2004; Durrleman et al., 2009) and it is sometimes referred to tangent PCA.

We briefly recall that, in standard PCA, the principal components of a dataset of *N* observations ***a***^*i*^ ∈ ℝ^*P*^ with *i* ∈ {1, …, *N*} are defined by the eigenvectors of the covariance matrix *C* with entries:

(21) $$C(i,j)=\frac{1}{N-1}{\left(a^{i}-\bar{a}\right)}^{t}(a^{j}-\bar{a})$$

with **a**^*i*^ given as a column vector, $\bar{a}=\frac{1}{N}\overset{N}{\underset{i=1}{\sum }}a^{i}$, and ***x***^*t*^ denotes the transposition of a vector ***x***.

In our case, our observations are initial momentum vectors **α**^*i*^ ∈ ℝ^3 × *n*^ and instead of computing the Euclidean scalar product in ℝ^3 × *n*^, we compute the scalar product with matrix *K*_*V*_, which is a natural choice since it corresponds to the inner product of the corresponding initial vector fields in the space *V*. The covariance matrix then writes:

(22) $$C_{V}(i,j)=\frac{1}{N-1}{\left(\alpha^{i}-\bar{\alpha }\right)}^{t}K_{V}(x)(\alpha^{j}-\bar{\alpha })$$

with $\bar{\alpha }$ the vector of the mean of momentum vectors, and ***x*** the vector of vertices of the template surface. We denote λ_1_, λ_2_, …, λ_*N*_ the eigenvalues of *C* in decreasing order, and ν^1^, ν^2^, …, ν^*N*^ the corresponding eigenvectors. The *k*-*th* principal mode is computed from the *k*-*th* eigenvector ν^*k*^ of *C*_*V*_, as follows:

(23) $$m^{k}=\bar{\alpha }+\sum_{j=1}^{N}\nu_{j}^{k}(\alpha^{j}-\bar{\alpha }).$$

The cumulative explained variance *CEV*_*k*_ for the *k* first principal modes is given by the equation:

(24) $$CEV_{k}=\frac{\sum_{h-1}^{k}\lambda_{h}}{\sum_{h-1}^{N}\lambda_{h}}$$

We can use geodesic shooting along any principal mode ***m***^*k*^ to visualize the corresponding deformations.

#### Remark

To analyse the population, we need to know the initial momentum vectors **α**^*i*^ which correspond to the matchings from the centroid to the subjects. For the IC1 and PW centroids, these initial momentum vectors were obtained by matching the centroid to each subject. For the IC2 centroid, since the mesh structure is composed of all vertices of the population, it is too computationally expensive to match the centroid toward each subject. Instead, from the deformation of each subject toward the centroid, we used the opposite vector of final momentum vectors for the analysis. Indeed, if we have two surfaces *S* and *T* and need to compute the initial momentum vectors from *T* to *S*, we can estimate the initial momentum vectors **α**^*TS*^(0) from *T* to *S* by computing the deformation from *S* to *T* and using the initial momentum vectors ${\tilde{\alpha }}^{TS}\left(0\right)=-\alpha^{ST}\left(1\right)$, which are located at vertices $\phi_{ST}\left(x^{S}\right)$.

### 4.2. Distance matrix approximation

Various methods such as Isomap (Tenenbaum et al., 2000) or Locally Linear Embedding (Roweis and Saul, 2000; Yang X. et al., 2011) use as input a matrix of pairwise distances between subjects. In the LDDMM setting, it can be computed using diffeomorphic distances: ρ(*S*_*i*_, *S*_*j*_) = *D*(*id*, φ_*ij*_). However, for large datasets, computing all pairwise deformation distance is computationally very expensive, as it involves *O*(*N*^2^) matchings. An alternative is to approximate the pairwise distance between two subjects through their matching from the centroid or template. This approach has been introduced in Yang X. F. et al. (2011). Here we use a first order approximation to estimate the diffeomorphic distance between two subjects:

(25) $$\tilde{\rho }(S_{i},S_{j})=\sqrt{\;\langle \alpha^{j}(0)-\alpha^{i}(0),K_{V}(x(0))(\alpha^{j}(0)-\alpha^{i}(0)\rangle },$$

with ***x***(0) the vertices of the estimated centroid or template and **α**^*i*^(0) is the vector of initial momentum vectors computed by matching the template to *S*_*i*_. Using such approximation allows to compute only *N* matchings instead of *N*(*N* − 1).

Note that ρ(*S*_*i*_, *S*_*j*_) is in fact the distance between *S*_*i*_ and φ_*ij*_(*S*_*i*_), and not between *S*_*i*_ and *S*_*j*_ due to the not exactitude of matchings. However we will refer to it as a distance in the following to denote the dissimilarity between *S*_*i*_ and *S*_*j*_.

## 5. Experiments and results

In this section, we evaluate the use of iterative centroids for statistical shape analysis. Specifically, we investigate the centring of the centroids within the population, their impact on population analysis based on Kernel PCA and on the computation of distance matrices. For our experiments, we used three different datasets: two real datasets and a synthetic one. In all datasets shapes are hippocampi. The hippocampus is an anatomical structure of the temporal lobe of the brain, involved in different memory processes.

### 5.1. Data

**Figure 2:**
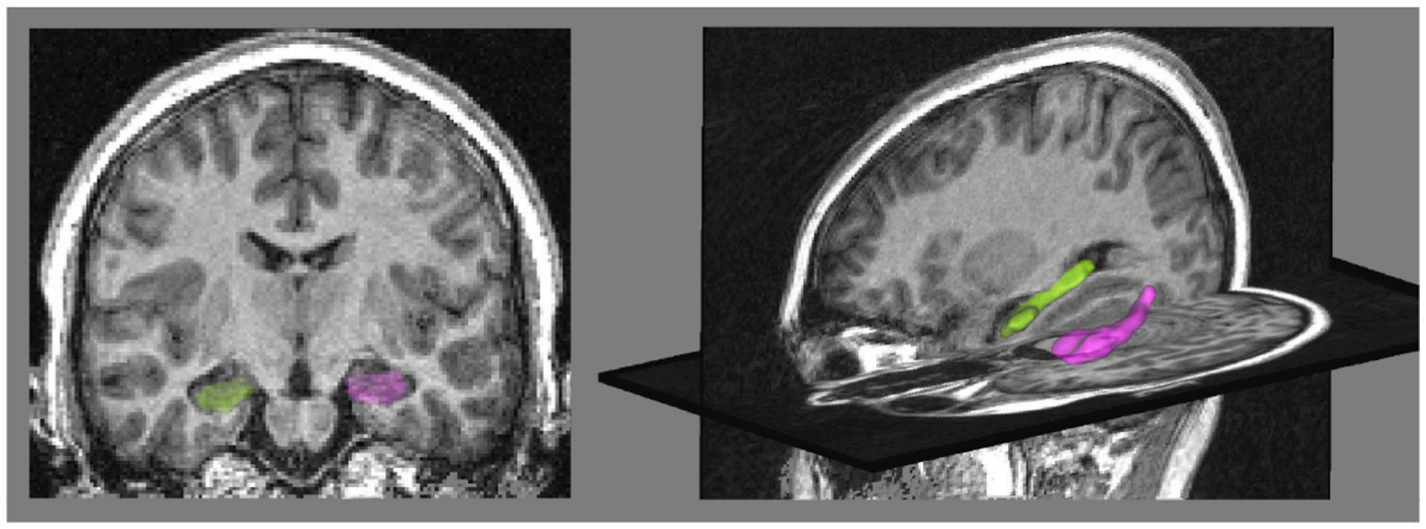
**(Left)** coronal view of the MRI with the binary masks of hippocampi segmented by the SACHA software (Chupin et al., 2009), the right hippocampus is in green and the left one in pink. **(Right)** 3D view of the hippocampus meshes.

The two real datasets are from the European database IMAGEN (Schumann et al., 2010) ^1^ composed of young healthy subjects. We segmented the hippocampi from T1-weighted Magnetic Resonance Images (MRI) of subjects using the SACHA software (Chupin et al., 2009) (see Figure 2). The synthetic dataset was built using deformations of a single hippocampal shape of the IMAGEN database.

#### 5.1.1. The synthetic dataset SD

SD is composed of synthetic deformations of a single shape *S*_0_, designed such that this single shape becomes the exact center of the population. We will thus be able to compare the computed centroids to this exact center. We generated 50 subjects for this synthetic dataset from *S*_0_, along geodesics in different directions. We randomly chose two orthogonal momentum vectors **β**_1_ and **β**_2_ in ℝ^3 × *n*^. We then computed momentum vectors **α**^*i*^,*i* ∈ {1, …, 25} of the form $k_{1}^{i}\beta_{1}+k_{2}^{i}\beta_{2}+k_{3}^{i}\beta_{3}$ with $\left(k_{1}^{i},k_{2}^{i},k_{3}^{i}\right)\in {\mathbb{R}}^{3},\forall i\in \left\{1,\ldots ,25\right\}$,$k_{j}^{i}\sim N\left(0,\sigma_{j}\right)$ with σ_1_ > σ_2_ ≫ σ_3_ and **β**_3_ a randomly selected momentum vector, adding some noise to the generated 2D space. We computed momentum vectors **α**^*j*^,*j* ∈ {26, …, 50} such as **α**^*j*^ = −**α**^*j*−25^. We generated the 50 subjects of the population by computing geodesic shootings of *S*_0_ using the initial momentum vectors **α**^*i*^,*i* ∈ {1, …, 50}. The population is symmetrical since $\overset{50}{\underset{i}{\sum }}\alpha^{i}=0$. It should be noted that all shapes of the dataset have the same mesh structure composed of *n* = 549 vertices. Data can be found here: https://doi.org/10.5281/zenodo.1420395.

#### 5.1.2. The real dataset RD50

RD50 is composed of 50 left hippocampi from the IMAGEN database. We applied the following preprocessing steps to each individual MRI. First, the MRI was linearly registered toward the MNI152 atlas, using the FLIRT procedure (Jenkinson et al., 2002) of the FSL software ^2^. The computed linear transformation was then applied to the binary mask of the hippocampal segmentation. A mesh of this segmentation was then computed from the binary mask using the BrainVISA software ^3^. All meshes were then aligned using rigid transformations to one subject of the population. For this rigid registration, we used a similarity term based on measures (as in Glaunès et al., 2004). All meshes were decimated in order to keep a reasonable number of vertices: meshes have on average 500 vertices.

#### 5.1.3. The real database RD1000

RD1000 is composed of 1,000 left hippocampi from the IMAGEN database. We applied the same preprocessing steps to the MRI data as for the dataset RD50. This dataset has also a score of Incomplete Hippocampal Inversion (IHI) (Cury et al., 2015), which is an anatomical variant of the hippocampus, present in 17% of the normal population.

### 5.2. Experiments

For the datasets SD and RD50 (which both contain 50 subjects), we compared the results of the three different iterative centroid algorithms (IC1, IC2, and PW). We also investigated the possibility of computing variational templates, initialized by the centroids, based on the approach presented in section 3.5. We could thus compare the results obtained when using the centroid directly to those obtained when using the most expensive (in term of computation time) template estimation. We thus computed 6 different centres: IC1, IC2, PW and the corresponding variational templates T(IC1), T(IC2), T(PW). For the synthetic dataset SD, we could also compare those 6 estimated centres to the exact centre of the population. For the real dataset RD1000 (with 1,000 subjects), we only computed the iterative centroid IC1.

For all computed centres and all datasets, we investigated: (1) the computation time; (2) whether the centres are close to a critical point of the Fréchet functional on the manifold discretised by the population; (3) the impact of the estimated centres on the results of Kernel PCA; (4) their impacts on approximated distance matrices.

Computation times are given for the different template estimation methods. For the variational template initialized with an estimated centroid, the computation time depends on the number of iteration needed to converge. We fixed this number to 5 *iter* (see section 3.5), which is usually, for the kind of shape we are using here, the number of iteration needed by the variational template to converge. For the variational template with standard initialisation (all objects of the population are considered, see section 3.5), we used 8 iterations.

To assess how close an estimated centre is to a critical point of the Fréchet functional, for the different centroids and variational templates, we computed a ratio *R* using the initial momentum vectors from centres to subjects. The ratio *R* takes values between 0 and 1:

(26) $$R=\frac{\|\frac{1}{N}\sum_{i=1}^{N}v^{i}(\cdot ,0){\|}_{V}}{\frac{1}{N}\sum_{i=1}^{N}∥v^{i}(\cdot ,0){\|}_{V}},$$

with *v*^*i*^(·, 0) the vector field of the deformation from the estimated centre to the subject *S*_*i*_, corresponding to the vector of initial momentum vectors **α**^*i*^(0). This ratio gives information about the centering of the centroid. In fact, in a pure riemannian setting, i.e., disregarding inaccuracies of matchings, and under some reasonable assumptions about curvature of the shape space, the Fréchet functional (see equation 18) would have a unique critical point corresponding to the Fréchet mean, which can be interpreted as the theoretical centre of the population. Now a null ratio *R* would mean that $\overset{N}{\underset{i=1}{\sum }}v^{i}\left(\cdot ,0\right)=0$, which is precisely the equation satisfied by critical points of the Fréchet functional. In practice, this ratio is expected to be close to zero but not null due to matchings inaccuracies.

We compared the results of Kernel PCA computed from these different centres by comparing the principal modes and the cumulative explained variance for different number of dimensions.

Finally, we compared the approximated distance matrices to the direct distance matrix.

For the RD1000 dataset, we will try to predict an anatomical variant of the normal population, the Incomplete Hippocampal Inversion (IHI), present in only 17% of the normal population.

### 5.3. Synthetic dataset SD

#### 5.3.1. Computation time

All the centroids and variational templates have been computed with σ_*V*_ = 15, which represents roughly half of the shapes length. Computation times for IC1 took 31 minu, 85 min for IC2, and 32 min for PW. The corresponding variational template initialized by these estimated centroids took 81 min (112 min in total), 87 min (172 min in total) and 81 min (113 min in total). As a reference, we also computed a template with the standard initialisation whose computation took **194** min. Computing a centroid saved between 56 and 84% of computation time over the template with standard initialization and between 50 and 72% over the template initialized by the centroid.

For the following analysis we need additional matching estimations to extract the initial momentum vectors from the mean shape to individual subjects. Those extra matchings are needed for tangent space analysis, and are not mandatory if one just wants to estimate a mean shape of a population (Cury et al., 2018). Estimating the initial momentum vectors from each centroids (IC1, IC2, and PW) added in average 22 min. No extra time for the variational template, since the method optimize those initial momentum vectors. Estimating initial momentum vectors and the centre of the population using IC1 took 53 min, using IC2 took 107 min and using PW took 54 min, which is still significantly faster than using a variational template.

#### 5.3.2. “Centring” of the estimated centres

Since in practice a computed centre is never at the exact centre, and its estimation may vary accordingly to the discretisation of the underlying shape space, we decided to generate another 49 synthetic populations as detailed in section 5.1, so we have 50 different discretisations of the shape space. For each of these populations, we computed the 3 centroids and the 3 variational templates initialized with these centroids. We calculated the ratio *R* described in the previous section for each estimated centre. Table 1 presents the mean and standard deviation values of the ratio *R* for each centroid and template, computed over these 50 populations.

**Table 1:** Synthetic dataset SD.

**Ratio**	***C***	***T*(*C*)**
IC1	0.07 ± 0.03	0.05 ± 0.02
IC2	0.07 ± 0.03	0.05 ± 0.02
PW	0.11 ± 0.05	0.07 ± 0.02

*Ratio R (equation 26) computed for the 3 centroids C, and the 3 variational templates initialized via these centroids (T(C))*.

In a pure Riemannian setting (i.e., disregarding the fact that matchings are not exact), a zero ratio would mean that we are at a critical point of the Fréchet functional, and under some reasonable assumptions on the curvature of the shape space in the neighborhood of the dataset (which we cannot check however), it would mean that we are at the Fréchet mean. By construction, the ratio computed from the exact centre using the initial momentum vectors **α**^*i*^ used for the construction of subjects *S*_*i*_ (as presented in section 5.1) is zero.

Ratios *R* are close to zero for all centroids and variational templates, indicating that they are close to the exact centre. Furthermore, the value of *R* may be partly due to the non-exactitude of the matchings between the estimated centres and the subjects. To become aware of this non-exactitude, we matched the exact centre toward all subjects of the SD dataset. The resulting ratio is *R* = 0.05. This is of the same order of magnitude as the ratios obtained in Table 1, indicating that the estimated centres are indeed very close to the exact centre.

#### 5.3.3. PCA on initial momentum vectors

We performed a PCA computed with the initial momentum vectors (see section 4 for details) from our different estimated centres (3 centroids, 3 variational templates and the exact centre).

We computed the cumulative explained variance for different number of dimensions of the PCA. Results are presented in Table 2. The cumulative explained variances are very similar for the different centres for any number of dimensions.

**Table 2:** Synthetic dataset SD.

	**1st mode**	**2nd mode**	**3rd mode**
Centre	0.829	0.989	0.994
IC1	0.829	0.990	0.995
IC2	0.833	0.994	0.996
PW	0.829	0.990	0.995
T(IC1)	0.829	0.995	0.999
T(IC2)	0.829	0.995	0.999
T(PW)	0.829	0.995	0.999

*Proportion of cumulative explained variance of kernel PCA computed from different centres*.

**Figure 3:**
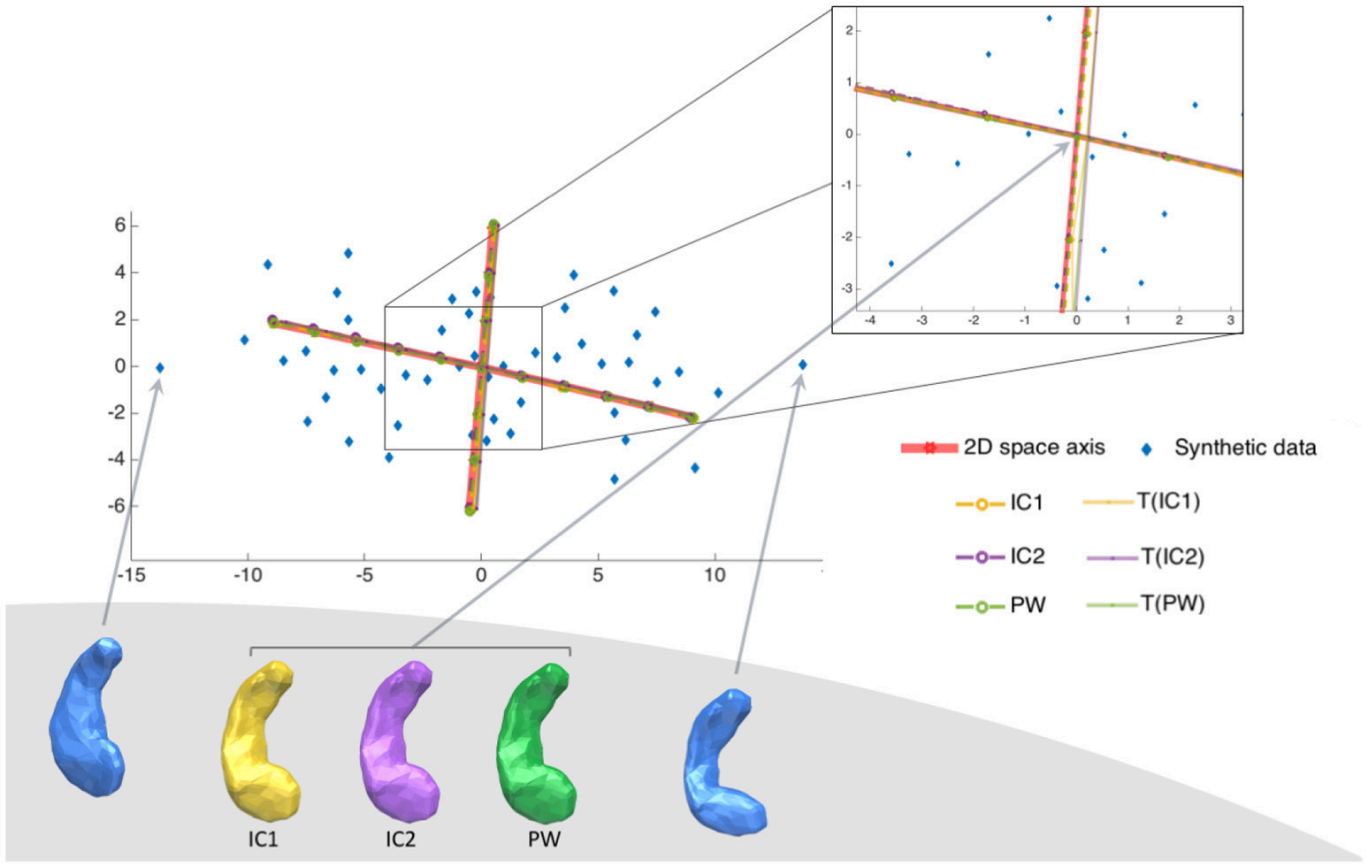
Synthetic dataset SD. Illustration of the two principal components of the 6 centres projected into the 2D space of the SD dataset. Synthetic population is in blue, and the real centre is at position (0,0). There is two principal components, projected into the 2D space of the population, computed from each of the 7 different estimated centres (marked in red for the exact centre, in yellow for IC1, in purple for IC2 and in green for PW). Each axis goes from −2σ to +2σ of the variability of the corresponding axe. This figure shows that the 6 different tangent spaces projected into the 2D space of shapes, are very similar, even if the centres have different positions.

We wanted to take advantage of the construction of this synthethic dataset to answer the question: Do the principal components explain the same deformations? The SD dataset allows to visualize the principal component relatively to the real position of the generator vectors and the population itself. Such visualization is not possible for real dataset since shape spaces are not generated by only 2 vectors. For this synthetic dataset, we can project principal components on the 2D space spanned by β_1_ and β_2_ as described in the previous paragraph. This projection allows displaying in the same 2D space subjects in their native space, and principal axes computed from the different Kernel PCAs. To visualize the first component (respectively the second one), we shot from the associated centre in the direction *k* * **m**^1^ (resp. **m**^2^) with $k\in \left[-2{\sqrt{\lambda }}_{1};+2{\sqrt{\lambda }}_{1}\right]$ (resp. ${\sqrt{\lambda }}_{2}$). Results are presented in Figure 3. The deformations captured by the 2 principal axes are extremely similar for all centres. The principal axes for the 7 centres, have all the same position within the 2D shape space. So for a similar amount of explained variance, the axes describe the same deformation.

Overall, for this synthetic dataset, the 6 estimated centres give very similar PCA results.

#### 5.3.4. Distance matrices

We then studied the impact of different centres on the approximated distance matrices. We computed the seven approximated distance matrices corresponding to the seven centres, and the direct pairwise distance matrix computed by matching all subjects to each other. Computation of the direct distance matrix took 1,000 min (17 h) for this synthetic dataset of 50 subjects. In the following, we denote as *aM*(*C*) the approximated distance matrix computed from the centre *C*.

**Figure 4:**
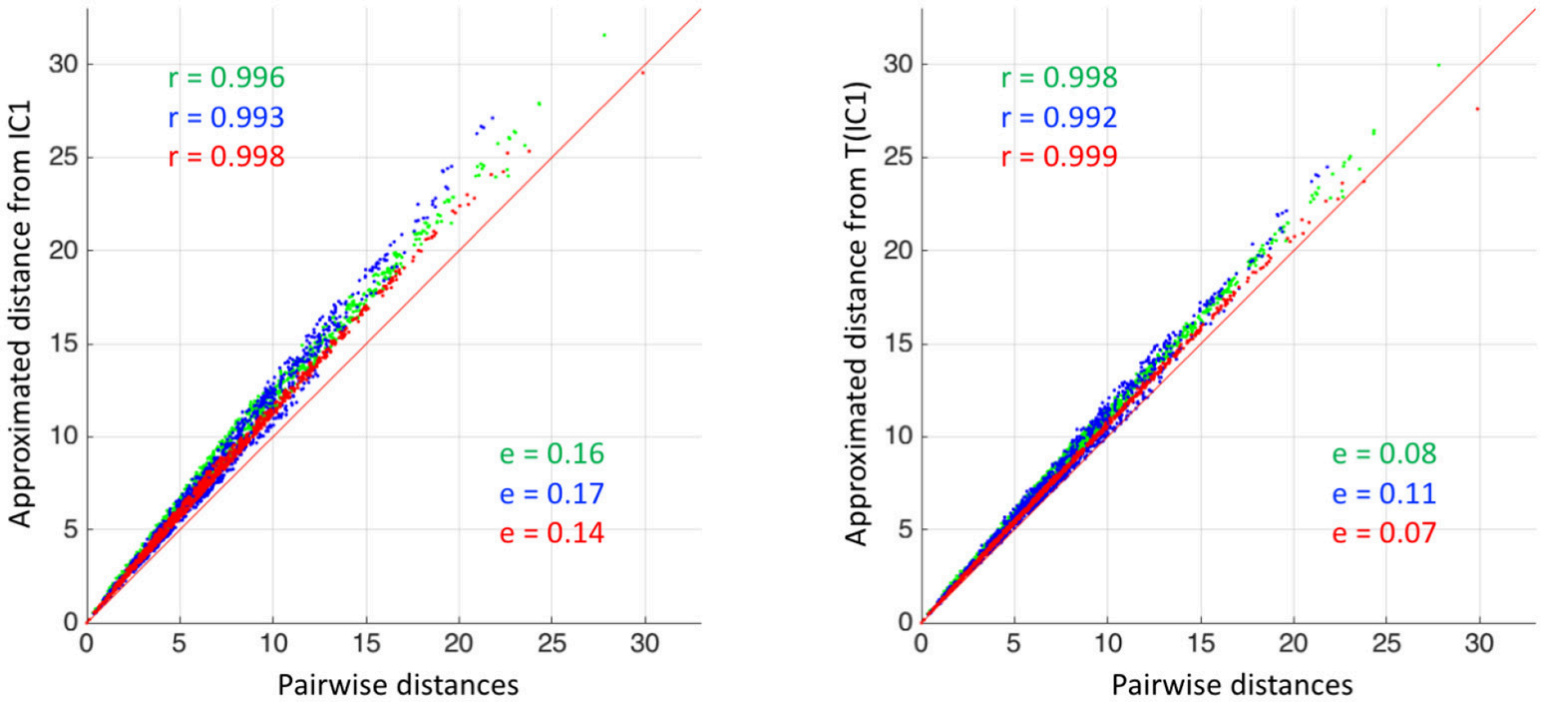
Synthetic dataset SD. **(Left)** Scatter plot between the approximated distance matrices *aM*(*IC*1) of 3 different populations, and the pairwise distances matrices of the corresponding populations. **(Right)** Scatter plot between the approximated distance matrices *aM*(*T*(*IC*1)) of 3 different populations, and the pairwise distances matrices of the corresponding populations. For both plots, the matrix error (equation 27) and Pearson correlation coefficients (only here to support the visualization of the scatter plots) are indicated in color. The red line corresponds to the identity.

To quantify the difference between these matrices, we used the following error *e*:

(27) $$e(M_{1},M_{2})=\frac{1}{N^{2}}\sum_{i,j=1}^{N}\frac{\left|M_{1}(i,j)-M_{2}(i,j)\right|}{max(M_{1}(i,j),M_{2}(i,j))}$$

with *M*_1_ and *M*_2_ two distance matrices. Results are reported in Table 3. For visualization of the errors against the pairwise computed distance matrices, we also computed the error between the direct distance matrix, by computing pairwise deformations (23h h of computation per population), for 3 populations randomly selected. Figure 4 shows scattered plots between the pairwise distances matrices and the approximated distances matrices of IC1 and T(IC1) for the 3 randomly selected populations. The errors between *aM*(*IC*1) matrices and the pairwise distance matrices of each of the populations are 0.17 0.16 and 0.14, respectively 0.11 0.08 and 0.07 for the errors with the corresponding *aM*(*T*(*IC*1)). We can observe a subtle curvature of the scatter-plot, which is due to the curvature of the shape space. This figure illustrates the good approximation of the distances matrices, regarding to the pairwise estimation distance matrix. The variational templates are getting slightly closer to the identity line, which is expected (as for the better ratio values) since they are extra iterations to converge toward a centre of the population, however the estimated centroids from the different algorithms, still provide a good approximation of the pairwise distances of the population. In conclusion for this set of synthetic population, the different estimated centres have also a little impact on the approximation of the distance matrices.

**Table 3:** Synthetic dataset SD.

***e*(*aM*(.), *aM*(.))**	**IC2**	**PW**	**T(IC1)**	**T(IC2)**	**T(PW)**
IC1	0.001 ± 0.001	0.002 ± 0.002	0.084 ± 0.02	0.084 ± 0.02	0.084 ± 0.02
IC2	0	0.003 ± 0.002	0.084 ± 0.02	0.084 ± 0.02	0.084 ± 0.02
PW		0	0.084 ± 0.02	0.084 ± 0.02	0.084 ± 0.02
T(IC1)			0	0.001 ± 0.001	0.002 ± 0.001
T(IC2)				0	0.002 ± 0.001

*Mean ± standard deviation errors e (equation 27) between the six different approximated distance matrices for each of the generated data sets*.

### 5.4. The real dataset RD50

We now present experiments on the real dataset RD50. For this dataset, the exact center of the population is not known, neither is the distribution of the population and meshes have different numbers of vertices and different connectivity structures.

#### 5.4.1. Computation time

We estimated our 3 centroids IC1 (75 min) IC2 (174 min) and PW (88 min), and the corresponding variational templates, which took respectively 188 , 252, and 183 min. The total computation time for *T*(*IC*1) is 263 min, 426 min for *T*(*IC*2) and 271 min for *T*(*PW*).

For comparison of computation time, we also computed a template using the standard initialization (the whole population as initialisation) which took 1220 min (20.3 h). Computing a centroid saved between 85 and 93% of computation time over the template with standard initialization and between 59 and 71% over the template initialized by the centroid.

Extra computation time was needed to estimate initial momentum vectors from IC1, IC2, and PW to each subject of the population, for subsequent analysis in the tangent spaces of each centres. Theses additional matchings took 19 min for IC1, 54 min for IC2, and 17 min for PW. The computation time is thus 94 min for IC1, 228 for IC2, and 105 min for PW which is considerably faster than estimating the variational template (1,220 min).

#### 5.4.2. Centring of the centres

As for the synthetic dataset, we assessed the centring of these six different centres. To that purpose, we first computed the ratio *R* of equation (26), for the centres estimated via the centroids methods, IC1 has a *R* = 0.25, for IC2 the ratio is *R* = 0.33 and for PW it is *R* = 0.32. For centres estimated via the variational templates initialized by those centroids, the ratio for T(IC1) is *R* = 0.21, for T(IC2) is *R* = 0.31 and for T(PW) is *R* = 0.26.

The ratios are higher than for the synthetic dataset indicating that centres are less centred. This was predictable since the population is not built from one surface via geodesic shootings as the synthetic dataset. In order to better understand these values, we computed the ratio for each subject of the population (after matching each subject toward the population), as if each subject was considered as a potential centre. For the whole population, the average ratio was 0.6745, with a minimum of 0.5543, and a maximum of 0.7626. These ratios are larger than the one computed for the estimated centres and thus the 6 estimated centres are closer to a critical point of the Frechet functional than any subject of the population.

#### 5.4.3. PCA on initial momentum vectors

As for the synthetic dataset, we performed six PCAs from the estimated centres.

**Figure 5:**
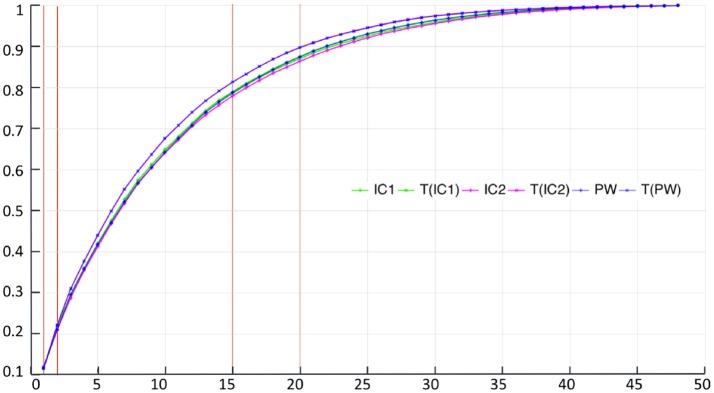
Real dataset RD50. Proportion of cumulative explained variance for Kernel PCAs computed from the 6 different centres, with respect to the number of dimensions. Vertical red lines indicate values displayed at Table 4.

Figure 5 and Table 4 show the proportion of cumulative explained variance for different number of modes. We can note that for any given number of modes, all PCAs result have really similar proportions of explained variance. The highest differences in cumulative explained variance, at a given number of component, between all 6 centres is 0.034 (>4% of the total variance) and it is between IC2 and T(PW) for the 20th mode of variation (see Table 4). To reach 30% of total variance, a KPCA computed from T(C) for any C ∈ {IC1, IC2, PW} needs 3 components while C needs 4 components. To reach 50% KPCA computed from all 6 centres need 7 components. Finally to reach 90% of total variance, T(C) needs 21 components while C needs 22.

**Table 4:** Real dataset RD50.

	**1st mode**	**2nd mode**	**15th mode**	**20th mode**
IC1	0.118	0.214	0.793	0.879
IC2	0.121	0.209	0.780	0.865
PW	0.117	0.209	0.788	0.875
T(IC1)	0.117	0.222	0.815	0.899
T(IC2)	0.115	0.220	0.814	0.898
T(PW)	0.116	0.221	0.814	0.898

*Proportion of cumulative explained variance for kernel PCAs computed from the 6 different centres, for different number of dimensions*.

#### 5.4.4. Distance matrices

As for the synthetic dataset, we then studied the impact of these different centres on the approximated distance matrices. A direct distance matrix was also computed (around 90 h of computation time). We compared the approximated distance matrices of the different centres to: (i) the approximated matrix computed with IC1; (ii) the direct distance matrix.

We computed the errors *e*(*M*_1_, *M*_2_) defined in equation 27. Results are presented in Table 5. Errors are small and with the same order of magnitude.

**Table 5:** Real dataset RD50.

	***e***(., ***aM***(***IC***1))		***e***(., ***dM***)	
	***C***	**T(*C*)**	***C***	**T(*C*)**
IC1	0	0.04	0.10	0.08
IC2	0.06	0.04	0.06	0.08
PW	0.03	0.04	0.08	0.07

*Errors e (equation 27) between the approximated distance matrices of each estimated centre and: (i) the approximated matrix computed with IC1 (left columns); (ii) the direct distance matrix (right columns)*.

**Figure 6:**
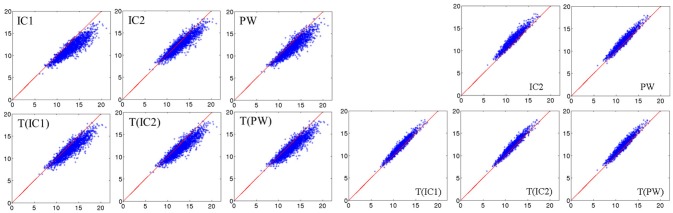
Real dataset RD50. **(Left)** 6 scatter plots between direct distance matrix and approximated distance matrices from the six centres. The red line corresponds to the identity. **(Right)** 5 scatter plots between the approximated distance matrix *aM*(*IC*1) computed from *IC*1 and the 5 others approximated distance matrices. The red line corresponds to the identity.

Figure 6-left shows scatter plots between the direct distance matrix and the six approximated distance matrices. Interestingly, we can note that the results are similar to those obtained by Yang X. F. et al. (2011), Figure 2). Figure 6-right shows scatter plots between the approximated distance matrix from IC1 and the five others approximated distance matrices. The approximated matrices thus seem to be largely independent of the chosen centre.

### 5.5. Real dataset RD1000

Results on the real dataset RD50 and the synthetic SD showed that results were highly similar for the 6 different centres. In light of these results and because of the large size of the real dataset RD1000, we only computed IC1 for this last dataset. The computation time was about 832 min (13.8 h) for the computation of the centroid using the algorithm IC1, and 12.6 h for matching the centroid to the population.

The ratio *R* of equation 26 computed from the IC1 centroid was 0.1011, indicating that the centroid is well centred within the population.

**Figure 7:**
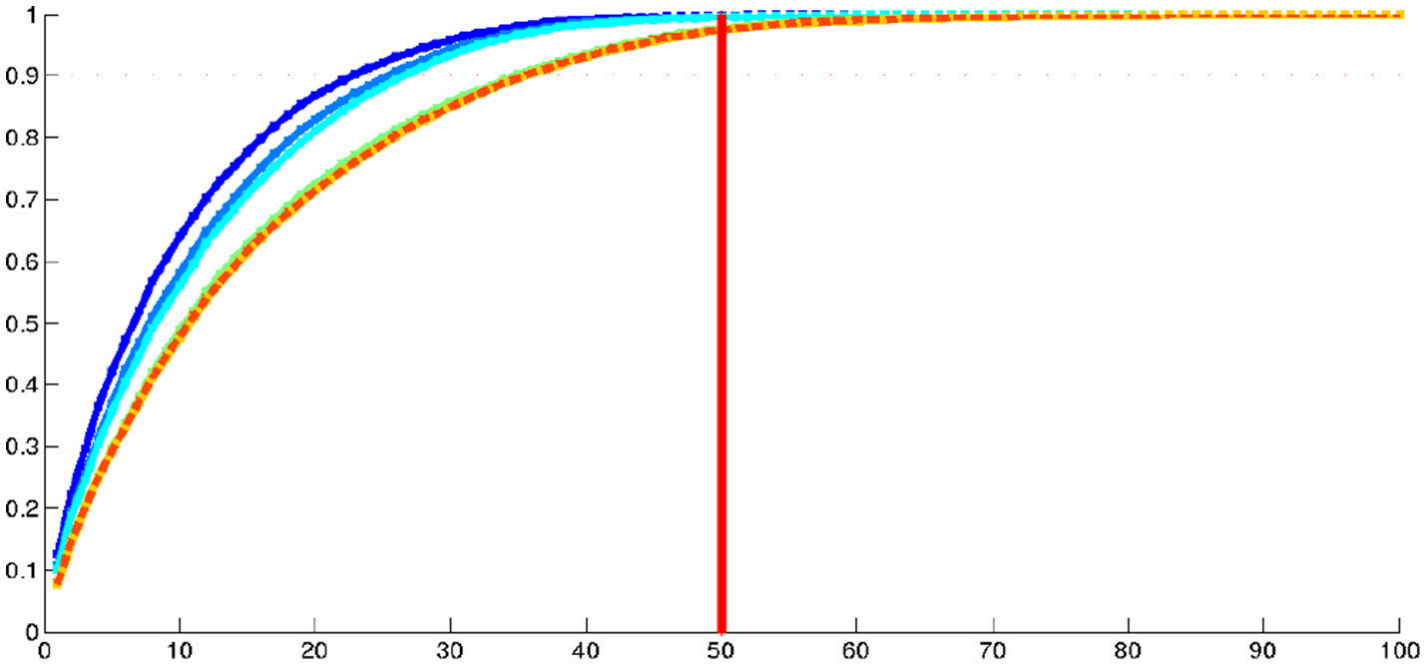
Real dataset RD1000. Proportion of cumulative explained variance of K-PCA as a function of the number of dimensions (in abscissa) and considering varying number of subjects. The dark blue curve was made using 100 subjects, the blue 200, the light blue 300, the green curve 500 subjects, the yellow one 800, very close to the dotted orange one which was made using 1,000 subjects.

We then performed a Kernel PCA on the initial momentum vectors from this IC1 centroid to the 1,000 shapes of the population. The proportions of cumulative explained variance from this centroid are 0.07 for the 1st mode, 0.12 for the 2nd mode, 0.48 for the 10th mode, 0.71 for the 20th mode, 0.85 for the 30th mode, 0.93 for the 40th mode, 0.97 for the 50th mode and 1.0 from the 100th mode. In addition, we explored the evolution of the cumulative explained variance when considering varying numbers of subjects in the analysis. Results are displayed in Figure 7. We can first note that about 50 dimensions are sufficient to describe the variability of our population of hippocampal shapes from healthy young subjects. Moreover, for large number of subjects, this dimensionality seems to be stable. When considering increasing number of subjects in the analysis, the dimension increases and converges around 50.

**Figure 8:**
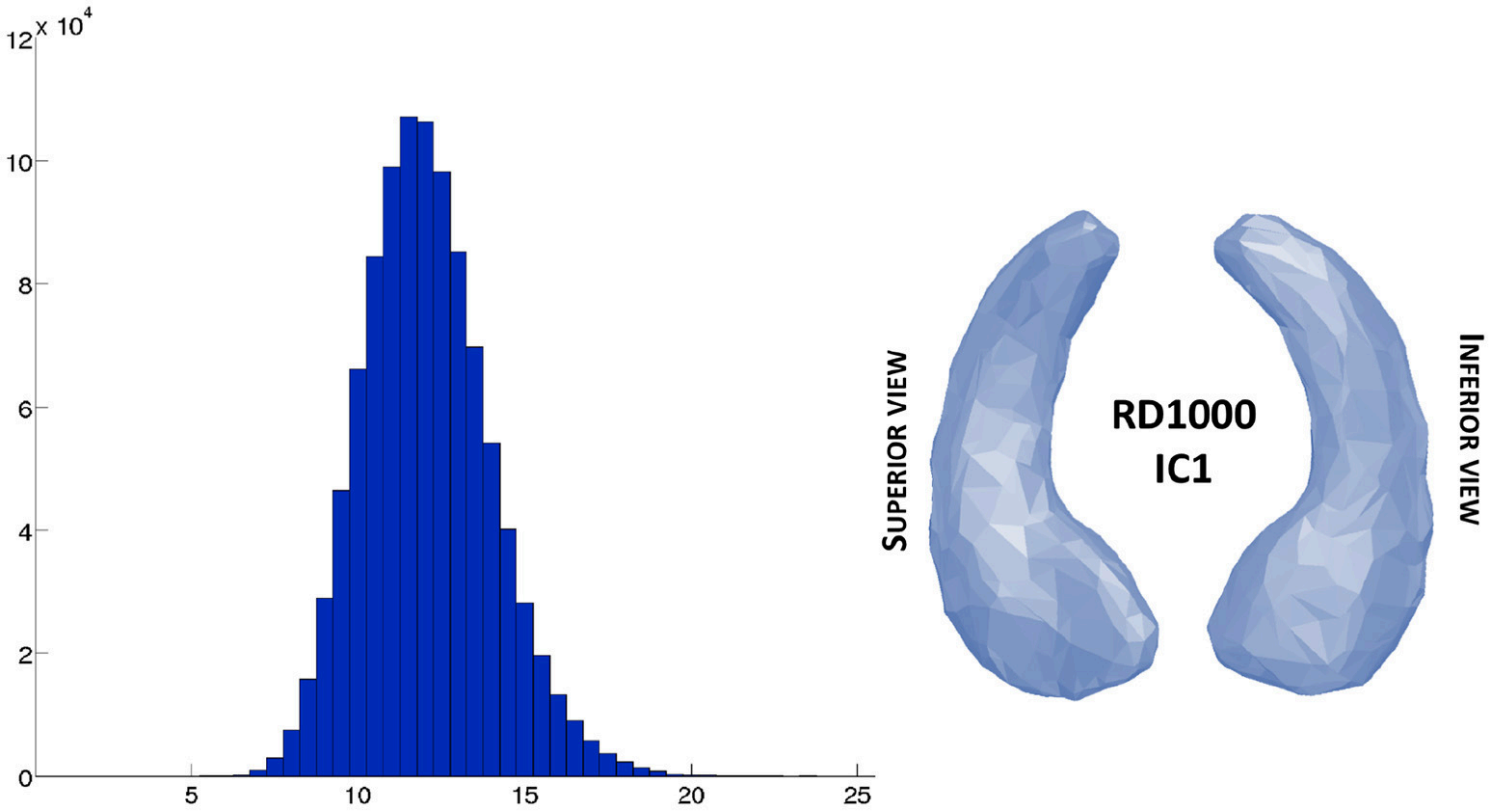
Real dataset RD1000. Left side shows histogram of the approximated distances of the large database RD1000 estimated from the computed centroid IC1 shown on the right side.

Finally, we computed the approximated distance matrix. Its histogram is shown in Figure 8. It can be interesting to note that, as for RD50, the average pairwise distance between the subject is around 12, which means nothing by itself, but the points cloud on Figure 6-left and the histogram on Figure 8, show no pairwise distances below 6, while the minimal pairwise distance for the SD50 dataset - generated by a 2D space - is zero. This corresponds to the intuition that, in a space of high dimension, all subjects are relatively far from each other.

#### 5.5.1. Prediction of IHI using shape analysis

Incomplete Hippocampal Inversion (IHI) is an anatomical variant of the hippocampal shape present in 17% of the normal population. All those 1,000 subjects have an IHI score (ranking from 0 to 8) (Cury et al., 2015), which indicates the strength of the incomplete inversion of the hippocampus. A null score means there is no IHI, 8 means there is a strong IHI. IHI scores were assessed on a T1 weighted MRI, in coronal view after registration of all MRI in the standard MNI space. The IHI score is based on the roundness of the hippocampal body, the medial positionning of the hippocampus within the brain, and the depth of the colateral sulcus and the occipito-temporal sulcus, which both are close to the hippocampus. Here we selected only hippocampi with good segmentation, we therefore removed strong IHI which could not be properly segmented. We now apply our approach to predict incomplete hippocampal inversions (IHI) from hippocampal shape parameters. Specifically, we predict the visual IHI score, which corresponds to the sum of the individual criteria as defined in Cury et al. (2015). Only 17% of the population have an IHI score higher than 3.5. We studied whether it is possible to predict the IHI score using statistical shape analysis on the RD1000 dataset composed of 1,000 healthy subjects (left hippocampus).

The deformation parameters characterizing the shapes of the population are the eigenvectors computed from the centroid IC1, and they are the independent variables we will use to predict the IHI scores. As we saw in the previous step, 40 eigenvectors are enough to explain 93% of the total anatomical variability of the population. We use the centred and normalized principal eigenvectors *X*_1, *i*_, …, *X*_40, *i*_ computed from the RD1000 database with *i* ∈ {1, …, 1000} to predict the IHI score *Y*. We simply used a multiple linear regression model (Hastie et al., 2009) which is written as $f\left(X\right)=\beta_{0}+\overset{40}{\underset{i=1}{\sum }}X_{i}\beta_{i}$ where β_0_, β_1_, …β_40_ are the regression coefficients to estimate. The standard method to estimate the regression coefficients is the least squares estimation method in which the coefficients β_*i*_ minimize the residual sum of squares $RSS\left(\beta \right)=\overset{N}{\underset{j=1}{\sum }}{\left(y_{j}-\beta_{0}-\overset{p}{\underset{i=1}{\sum }}x_{ji}\beta_{i}\right)}^{2}$, which leads to the estimated $\hat{\beta }$ (with matrix notations) $\hat{\beta }={\left(X^{T}X\right)}^{-1}X^{T}Y$. For each number of dimensions *p* ∈ {1, …, 40} we validated the quality of the computed model with the adjusted coefficient of determination $R_{adj}^{2}$, which expresses the part of explained variance of the model with respect to the total variance:

(28) $$R_{adj}^{2}=1-\frac{SSE/(N-p)}{SST/(N-1)}$$

with $SSE=\overset{N}{\underset{i}{\sum }}{\left(y_{i}-\left(X_{1\ldots p,i}^{T}\hat{\beta }\right)\right)}^{2}$ the residual variance due to the model and $SST=\overset{N}{\underset{i=1}{\sum }}{\left(y_{i}-Ȳ\right)}^{2}$ the total variance of the model. The $R_{adj}^{2}$ coefficient, unlike *R*^2^, takes into account the number of variables and therefore does not increase with the number of variables. One can note that *R* is the coefficient of correlation of Pearson. We then tested the significance of each model by computing the F statistic

(29) $$F=\frac{R^{2}/p}{\left(1-R^{2})/(N-p-1\right)}$$

which follows a F-distribution with (*p, n*−*p*−1) degrees of freedom. So for each number of variables (i.e., dimensions of the PCA space) we computed the adjusted coefficient of determination to evaluate the model and the *p*-value to evaluate the significance of the model.

Then we used the *k*-fold cross validation method which consists in using 1000−*k* subjects to predict the *k* remaining ones. To quantify the prediction of the model, we used the traditional mean square error *MSE* = *SSE*/*N* which corresponds to the unexplained residual variance. For each model, we computed 10,000 *k*-fold cross validation and displayed the mean and the standard deviation of *MSE* corresponding to the model.

**Figure 9:**
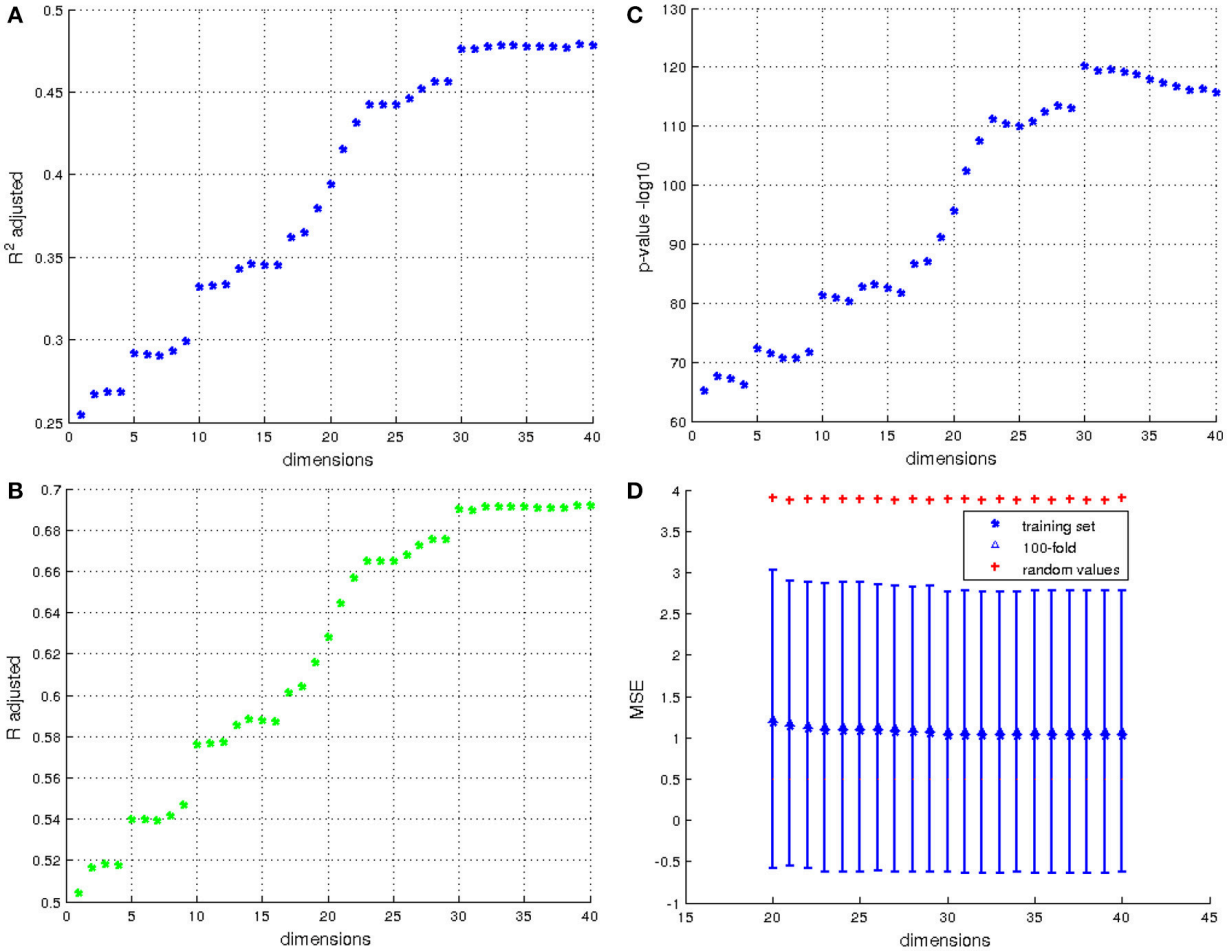
Results for prediction of IHI scores. **(A)** Values of the adjusted coefficient of determination using from 1 to 40 eigenvectors resulting from the PCA. **(B)** the coefficient correlation corresponding to the coefficient of determination of A. **(C)** The *p*-values in −*log*_10_ of the corresponding coefficient of determination. **(D)** Cross validation of the models using 20 to 40 dimensions by 100-fold. The red cross indicates the *MSE* of the model predicted using random values, and the errorbar corresponds to the standard deviation of *MSE* computed from 10,000 cross validations for each model, the triangle corresponds to the average *MSE*.

Results are given at Figure 9, and display the coefficient of determination of each model. The cross validation is only computed on models with a coefficient of correlation higher than 0.5, so models using at least 20 dimensions. For the *k*-fold cross validation, we chose *k* = 100 which represents 10% of the total population. Figure 9D presents results of cross validation; for each model computed from 20 to 40 dimensions we computed the mean of the 10,000 *MSE* of the 100-fold and its standard deviation. To have a point of comparison, we also computed the *MSE* between the IHI scores and random values which follow a normal distribution with the same mean and standard deviation as the IHI scores (red cross on the Figure). The *MSE* of the cross validation are similar to the *MSE* of the training set. This result shows that using the first 30 to 40 principal components of initial momentum vectors computed from a centroid of the population, it is possible to predict the IHI score with a correlation of 69%. The firsts principal components (between 1 and 20) represent general variance maybe characteristic of the normal population, the shape differences related to IHI appear after. It is indeed expected that the principal (i.e., the first once) modes of variation does not capture a modification of the anatomy present in only 17% of the population.

## 6. Discussion and conclusion

In this paper, we proposed a method for template-based shape analysis using diffeomorphic centroids. This approach leads to a reasonable computation time making it applicable to large datasets. It was thoroughly evaluated on different datasets including a large population of 1,000 subjects. Codes can be found here: https://github.com/cclairec/Iterative_Centroid and synthetic data can be found here: https://doi.org/10.5281/zenodo.1420395.

The results demonstrate that the method adequately captures the variability of the population of hippocampal shapes with a reasonable number of dimensions. In particular, Kernel PCA analysis showed that the large population of left hippocampi of young healthy subjects can be explained, for the metric we used, by a relatively small number of variables (around 50). Moreover, when a large enough number of subjects was considered, the number of dimensions was independent of the number of subjects.

The comparisons performed on the two small datasets show that the different centroids or variational templates lead to very similar results. This can be explained by the fact that in all cases the analysis is performed on the tangent space to the template, which correctly approximates the population in the shape space. Moreover, we showed that the different estimated centres are all close to the Frechet mean of the population.

While all centres (centroids or variational templates) yield comparable results, they have different computation times. IC1 and PW centroids are the fastest approaches and can save between 70 and 90% of computation time over the variational template. Thus, for the study of hippocampal shape, IC1 or PW algorithms seem to be more adapted than IC2 or the variational template estimation. However, it is not clear whether the same conclusion would hold for more complex sets of anatomical structures, such as an exhaustive description of cortical sulci (Auzias et al., 2011). Besides, as mentioned above in section 5.3, unlike with the variational template estimation, centroid computations do not directly provide transformations between the centroid and the population which must be computed afterwards to obtain momentum vectors. This requires *N* more matchings, which almost doubles the computation time. Even with this additional step, centroid-based shape analysis stills leads to a competitive computation time (about 26 h for the complete procedure on the large dataset of 1,000 subjects).

Here we computed for all our datasets the initial momentum vectors from the mean shape to each subject of the population to apply a kernel PCA on the mean shape. But this is not a mandatory step, for example, in Cury et al. (2018) the iterative centroid method have been applied to provide a mean shape used afterwards as a baseline shape for an other analysis. It is also possible, for visualization purposes, to estimate different templates within a population (controls vs. patients for example) and identify where shape differences are located between templates.

In future work, this approach could be improved by using a discrete parametrisation of the LDDMM framework (Durrleman et al., 2011), based on a finite set of control points. The control points number and position are independent from the shapes being deformed as they do not require to be aligned with the shapes' vertices. Even if the method accepts any kind of topology, for more complex and heavy meshes like the cortical surface (which can have more than 20000 vertices per subjects), we could also improve the method presented here by using a multiresolution approach (Tan and Qiu, 2016). An other interesting point would be to study the impact of the choice of parameters on the number of dimensions needed to describe the variability population (in this study the parameters were selected to optimize the matchings). Finally we can note that this template-based shape analysis can be extended to data types such as images or curves.

## Ethics statement

London: Psychiatry, Nursing and Midwifery (PNM) Research Ethics Subcommittee (RESC), Waterloo Campus, Kings College London. Nottingham: University of Nottingham Medical School Ethics Committee. Mannheim: Medizinische Fakultaet Mannheim, Ruprecht Karl Universitaet Heidelberg and Ethik-Kommission II an der Fakultaet fuer Kliniksche Medizin Mannheim. Dresden: Ethikkommission der Medizinischen Fakultaet Carl Gustav Carus, TU Dresden Medizinische Fakultaet. Hamburg: Ethics board, Hamburg Chamber of Phsyicians. Paris: CPP IDF VII (Comit de protection des personnes Ile de France), ID RCB: 2007-A00778-45 September 24th 2007. Dublin: TCD School of Psychology REC. Berlin: ethics committee of the Faculty of Psychology. And Mannheims ethics committee approved the whole study.

## Author contributions

CC guarantor of integrity of entire study. Study concepts and design or data acquisition or data analysis and interpretation, all authors; manuscript drafting or manuscript revision for important intellectual content, all authors; approval of final version of submitted manuscript, all authors. CC, JG, and OC literature research. MC, RT, GS, VF, and J-BP Clinical studies and pre-processings. CC, JG, RT, MC, and OC Statistical analysis and manuscript editing, all authors.

### Conflict of interest statement

The authors declare that the research was conducted in the absence of any commercial or financial relationships that could be construed as a potential conflict of interest.

## Footnotes

^1^http://www.imagen-europe.com/

^2^http://fsl.fmrib.ox.ac.uk/fsl/fslwiki/FslOverview

^3^http://brainvisa.info/web/index.html

## References

[B1] Afsari B. (2011). Riemannian Lp center of mass: Existence, uniqueness, and convexity. Proc. Am. Math. Soc. 139, 655–673. 10.1090/S0002-9939-2010-10541-5

[B2] Afsari B. Tron R. Vidal R. (2013). On the convergence of gradient descent for finding the Riemannian center of mass. SIAM J. Control Optimizat. 51, 2230–2260. 10.1137/12086282X

[B3] Arnaudon M. Dombry C. Phan A. Yang L. (2012). Stochastic algorithms for computing means of probability measures. Stochast. Process. Their Appl. 122, 1437–1455. 10.1016/j.spa.2011.12.011

[B4] Ashburner J. Hutton C. Frackowiak R. Johnsrude I. Price C. Friston K.. (1998). Identifying global anatomical differences: deformation-based morphometry. Hum. Brain Mapp. 6, 348–357. 9788071 10.1002/(SICI)1097-0193(1998)6:5/6<348::AID-HBM4>3.0.CO;2-P PMC6873376

[B5] Auzias G. Colliot O. Glaunes J. A. Perrot M. Mangin J.-F. Trouvé A.. (2011). Diffeomorphic brain registration under exhaustive sulcal constraints. Med. Imaging IEEE Trans. 30, 1214–1227. 10.1109/TMI.2011.2108665 21278014

[B6] Baulac M. Grissac N. D. Hasboun D. Oppenheim C. Adam C. Arzimanoglou A.. (1998). Hippocampal developmental changes in patients with partial epilepsy: Magnetic resonance imaging and clinical aspects. Ann. Neurol. 44, 223–233. 9708545 10.1002/ana.410440213

[B7] Beg M. F. Miller M. I. Trouvé A. Younes L. (2005). Computing large deformation metric mappings via geodesic flows of diffeomorphisms. Int. J. Comput. Vision 61, 139–157. 10.1023/B:VISI.0000043755.93987.aa

[B8] Charon N. Trouvé A. (2013). The varifold representation of nonoriented shapes for diffeomorphic registration. SIAM J. Imaging Sci. 6, 2547–2580. 10.1137/130918885

[B9] Chung M. Worsley K. Paus T. Cherif C. Collins D. Giedd J.. (2001). A unified statistical approach to deformation-based morphometry. NeuroImage 14, 595–606. 10.1006/nimg.2001.0862 11506533

[B10] Chupin M. Hammers A. Liu R. S. N. Colliot O. Burdett J. Bardinet E.. (2009). Automatic segmentation of the hippocampus and the amygdala driven by hybrid constraints: method and validation. NeuroImage 46, 749–761. 10.1016/j.neuroimage.2009.02.013 19236922 PMC2677639

[B11] Colle R. Cury C. Chupin M. Deflesselle E. Hardy P. Nasser G.. (2016). Hippocampal volume predicts antidepressant efficacy in depressed patients without incomplete hippocampal inversion. NeuroImage Clin. 12(Suppl. C), 949–955. 10.1016/j.nicl.2016.04.009 27995060 PMC5153557

[B12] Cury C. (2015). Statistical Shape Analysis of the Anatomical Variability of the Human Hippocampus in Large Populations. Theses, Université Pierre et Marie Curie, Paris VI.

[B13] Cury C. Durrleman S. Cash D. Lorenzi M. Nicholas J. Bocchetta M. (2018). Spatiotemporal analysis for detection of pre-symptomatic shape changes in neurodegenerative diseases: applied to GENFI study. Biorxiv[Pre print]. 10.1101/385427 PMC6414401 30529631

[B14] Cury C. Glaunès J. Chupin M. Colliot O. (2017). Analysis of anatomical variability using diffeomorphic iterative centroid in patients with Alzheimer's disease. Comput. Methods Biomech. Biomed. Eng. Imaging Visual. 5, 350–358. 10.1080/21681163.2015.1035403

[B15] Cury C. Glaunès J. A. Chupin M. Colliot O. (2014a). “Fast template-based shape analysis using diffeomorphic iterative centroid,” in MIUA 2014-Medical Image Understanding and Analysis 2014 (London, UK: Constantino Carlos Reyes-Aldasoro and Gref Slabaugh), 39–44.

[B16] Cury C. Glaunès J. A. Colliot O. (2013). “Template estimation for large database: a diffeomorphic iterative centroid method using currents,” in GSI Vol. 8085, Lecture Notes in Computer Science, eds F. Nielsen and F. Barbaresco (Paris: Springer), 103–111.

[B17] Cury C. Glaunès J. A. Colliot O. (2014b). “Diffeomorphic iterative centroid methods for template estimation on large datasets,” in Geometric Theory of Information, Signals and Communication Technology, ed F. Nielsen (Springer International Publishing), 273–299.

[B18] Cury C. Toro R. Cohen F. Fischer C. Mhaya A. Samper-González J.. (2015). Incomplete Hippocampal Inversion: A Comprehensive MRI Study of Over 2000 Subjects. Front. Neuroanat. 9:160. 10.3389/fnana.2015.00160 26733822 PMC4686650

[B19] de Rham G. (1955). Variétés différentiables. Formes, courants, formes harmoniques. Hermann: Inst. Math. Univ. Nancago III.

[B20] Durrleman S. (2010). Statistical Models of Currents for Measuring the Variability of Anatomical Curves, Surfaces and Their Evolution. Ph. D. thesis, University of Nice-Sophia Antipolis.

[B21] Durrleman S. Pennec X. Trouvé A. Ayache N. (2008). “A forward model to build unbiased atlases from curves and surfaces,” in 2nd Medical Image Computing and Computer Assisted Intervention. Workshop on Mathematical Foundations of Computational Anatomy (New York, NY), 68–79.

[B22] Durrleman S. Pennec X. Trouvé A. Ayache N. (2009). Statistical models of sets of curves and surfaces based on currents. Med. Image Anal. 13, 793–808. 10.1016/j.media.2009.07.007 19679507

[B23] Durrleman S. Prastawa M. Gerig G. Joshi S. (2011). Optimal data-driven sparse parameterization of diffeomorphisms for population analysis. In eds D. Hutchison, T. Kanade, J. Kittler, J. M. Kleinberg, F. Mattern, J. C. Mitchell, et al , *Information Processing in Medical Imaging, volume 6801* (Berlin; Heidelberg: Springer Berlin Heidelberg), 123–134. 10.1007/978-3-642-22092-0_11 PMC3758258 21761651

[B24] Glaunès J. (2005). Transport Par Difféomorphismes de Points, de Mesures et de Courants Pour la Comparaison de Formes et l'anatomie numérique. Ph. D. thesis, Université Paris 13.

[B25] Glaunès J. and Joshi, S. (2006). “Template estimation from unlabeled point set data and surfaces for Computational Anatomy,” in Proceedings of the International Workshop on the Mathematical Foundations of Computational Anatomy (MFCA-2006) eds X. Pennec and S. Joshi (Copenhagen: LNCS Springer) 29–39.

[B26] Glaunès J. Trouve A. Younes L. (2004). “Diffeomorphic matching of distributions: a new approach for unlabelled point-sets and sub-manifolds matching,” in Computer Vision and Pattern Recognition, 2004. CVPR 2004, Vol. 2, II–712–II–718.

[B27] Hastie T. Tibshirani R. Friedman J. Hastie T. Friedman J. Tibshirani R. (2009). The Elements of Statistical Learning. Springer.

[B28] Jenkinson M. Bannister P. Brady M. Smith S. (2002). Improved optimization for the robust and accurate linear registration and motion correction of brain images. Neuroimage 17, 825–841. 10.1016/S1053-8119(02)91132-8 12377157

[B29] Karcher H. (1977). Riemannian center of mass and mollifier smoothing. Commun. Pure Appl. Math. 30, 509–541.

[B30] Kendall W. S. (1990). Probability, convexity, and harmonic maps with small image I: uniqueness and fine existence. Proc. Lond. Math. Soc. 3, 371–406.

[B31] Le H. (2004). Estimation of Riemannian barycentres. LMS J. Comput. Math. 7, 193–200. 10.1112/S1461157000001091

[B32] Lorenzi M. (2012). Deformation-Based Morphometry of the Brain for the Development of Surrogate Markers in Alzheimer's Disease. Ph. D. thesis, Université de Nice Sophia-Antipolis.

[B33] Ma J. Miller M. I. Trouvé A. Younes L. (2008). Bayesian template estimation in computational anatomy. NeuroImage 42. 252–261. 10.1016/j.neuroimage.2008.03.056 18514544 PMC2602958

[B34] Roweis S. T. and Saul, L. K. (2000). Nonlinear dimensionality reduction by locally linear embedding. Science 290, 2323–2326. 10.1126/science.290.5500.2323 11125150

[B35] Schölkopf B. Smola A. Müller K.-R. (1997). “Kernel principal component analysis,” in Artificial Neural Networks—ICANN'97 (Lausanne: Springer), 583–588.

[B36] Schumann G. Loth E. Banaschewski T. Barbot A. Barker G. Büchel C.. (2010). The IMAGEN study: reinforcement-related behaviour in normal brain function and psychopathology. Mol Psychiatry 15, 1128–1139. 10.1038/mp.2010.4 21102431

[B37] Schwartz L. (1952). Théorie des distributions. Bull. Amer. Math. Soc. 58 , 78-85.

[B38] Tan M. and Qiu, A. (2016). Large deformation multiresolution diffeomorphic metric mapping for multiresolution cortical surfaces: a coarse-to-fine approach. IEEE Transactions on Image Processing, 25, 4061–4074. 10.1109/TIP.2016.2574982 27254865

[B39] Tenenbaum J. Silva V. Langford J. (2000). A global geometric framework for nonlinear dimensionality reduction. Science 290, 2319–2323. 10.1126/science.290.5500.2319 11125149

[B40] Trouvé A. (1998). Diffeomorphisms groups and pattern matching in image analysis. Int. J. Comput. Vision 28, 213–221.

[B41] Vaillant M. and Glaunès, J. (2005). “Surface matching via currents,” in Information Processing in Medical Imaging Glenwood (Springs, CO: Springer), 381–392. 10.1007/11505730_32 17354711

[B42] Vaillant M. Miller M. I. Younes L. Trouvé A. (2004). Statistics on diffeomorphisms via tangent space representations. NeuroImage 23, S161–S169. 10.1016/j.neuroimage.2004.07.023 15501085

[B43] Von Luxburg U. (2007). A tutorial on spectral clustering. Stat. Comput. 17, 395–416. 10.1007/s11222-007-9033-z

[B44] Yang X. Goh A. Qiu A. (2011). Locally Linear Diffeomorphic Metric Embedding (LLDME) for surface-based anatomical shape modeling. NeuroImage 56, 149–161. 10.1016/j.neuroimage.2011.01.069 21281721

[B45] Yang X. F. Goh A. Qiu A. (2011). Approximations of the diffeomorphic metric and their applications in shape learning. Inf. Proc. Med. Imaging 22, 257–270. 10.1007/978-3-642-22092-0_22 21761662

[B46] Younes L. (2010). Shapes and Diffeomorphisms, Vol. 171. Springer.

